# NSTracker: 3-Axis Antenna Control Software for Stable Satellite Data Acquisition

**DOI:** 10.3390/s26175477

**Published:** 2026-08-29

**Authors:** Euteum Choi, Mingeun Cho, Seungmin Tak, Seongjin Lee

**Affiliations:** 1 Defense and Technology Convergence Institute (DTCI), Gyeongsang National University, 501 Jinju-Daero, Jinju-si 52828, Gyeongsangnam-do, Republic of Korea; etchoi@gnu.ac.kr; 2 Nsquare Co., Ltd., 501 Jinju-Daero, Jinju-si 52828, Gyeongsangnam-do, Republic of Korea; mgcho@nsqur.com (M.C.); smtak@nsqur.com (S.T.); 3 Department of AI Convergence Engineering, Gyeongsang National University, 501 Jinju-Daero, Jinju-si 52828, Gyeongsangnam-do, Republic of Korea

**Keywords:** satellite communication, satellite ground stations, antenna pointing system (ASP), gimbal lock problem

## Abstract

With the rapid growth of the New Space era, the increasing number and diversity of Low Earth Orbit (LEO) satellites demand generic and reusable antenna control software capable of stable and accurate tracking across heterogeneous orbital and antenna environments. Previous works have primarily focused on two-axis antenna systems, relying on step-based tracking or Two-Line Element (TLE)-based orbit prediction. However, such approaches suffer from manual initialization requirements and gimbal lock at high elevation angles, limiting continuous tracking performance. Although three-axis antenna structures have been proposed to address these issues, their lack of software-level generality and experimental validation restricts practical reuse across antenna platforms. This paper presents a generic three-axis parabola antenna control software that integrates TLE-based orbit prediction, a gimbal lock avoidance algorithm, and an antenna specification reflection function. By decoupling antenna-specific parameters from the core tracking logic, the proposed system enables flexible adaptation to diverse antenna configurations. Experimental validation using a real three-axis antenna and simulations demonstrates high tracking accuracy, achieving an average gain error within 0.466 dBm for GEO-KOMPSAT-2A and stable signal strength above 55 dBm for the LEO satellite ARIRANG-5. Furthermore, analysis of 11,469 gimbal-lock-prone satellites confirms robust avoidance performance across diverse orbital conditions.

## 1. Introduction

With the advent of the New Space era, private companies are rapidly developing and deploying Low Earth orbit (LEO) satellites, expanding satellite-based services across defense, agriculture, meteorology, and remote sensing [1]. As satellite constellations grow in scale and diversity, ground stations play an increasingly important role in reliable data acquisition and timely processing [2,3]. A ground station fundamentally consists of a satellite antenna and an antenna control system responsible for satellite tracking [4]. Although antenna hardware continues to advance, tracking performance is largely determined by control software that performs orbit prediction, coordinate transformation, and real-time motion control. Therefore, generic and reusable antenna control software capable of operating across different antenna configurations and orbital environments is increasingly required.

Parabola antennas are widely used in ground stations because of their high gain and narrow beamwidth. However, conventional two-axis systems suffer from gimbal lock at high elevation angles, limiting continuous satellite tracking. Introducing an additional mechanical axis can mitigate this limitation, but three-axis configurations increase mechanical complexity, and their control strategies are often dependent on antenna geometry, actuator arrangement, and operational constraints [5]. Moreover, the additional axis introduces multiple feasible pointing attitudes, requiring the controller to select a stable solution while considering axis coupling, cable-wrap limits, angular velocity constraints, and satellite motion. Without an appropriate software-level avoidance strategy, discontinuous motion or excessive axis rotation may still occur during high-elevation tracking.

Antenna tracking methods are generally classified into closed-loop approaches based on received signal feedback and open-loop approaches based on orbital prediction [6]. Closed-loop methods depend on signal conditions and operator intervention, whereas open-loop methods using TLE (Two-Line Element set) and orbit propagation models can provide deterministic and repeatable tracking. These characteristics make open-loop control particularly suitable for automated and scalable ground station operation.

Previous works have proposed various approaches to improve tracking accuracy and continuity. Step-based methods maximize received signal strength but require manual initialization and may suffer from side-lobe tracking [7]. TLE- and SGP4-based approaches reduce tracking loss but can exhibit discontinuities near high-elevation regions when gimbal lock is not explicitly considered [8]. Multi-axis antenna systems have also been introduced to mitigate gimbal lock, but their control algorithms are often tightly coupled to specific antenna geometries, limiting portability across heterogeneous platforms [9].

To address these limitations, this paper presents a software-centric control framework for three-axis parabola antennas that integrates TLE-based orbit prediction, gimbal lock avoidance, and configurable antenna specifications. Antenna-dependent properties, including axis configuration, motion range, cable-wrap limits, angular velocity limits, and initial reference posture, are separated from the core orbit prediction, coordinate transformation, and singularity avoidance algorithms. This separation enables the same control logic to be reused across different three-axis antenna platforms without redesigning the tracking algorithms.

The novelty of this work lies in formulating multi-axis satellite tracking as a software generalization problem rather than as a hardware-specific solution. The presented software is validated using an actual three-axis parabola antenna and simulation environments. Experimental results demonstrate accurate and continuous tracking in GEO and LEO scenarios while supporting flexible adaptation to different antenna specifications.

This paper is organized as follows. Section 2 presents the background, and Section 3 reviews related work. Section 4 describes the system architecture and key algorithms. Section 5 presents the experimental setup and results. Finally, Section 6 concludes the paper and discusses future research directions.

## 2. Background

Satellite antennas are essential components for transmitting and receiving satellite signals, and common types include helical, horn, phased array, and parabola antennas. Helical and horn antennas offer relatively simple structures and wide frequency characteristics but have limitations in gain, whereas phased array antennas provide electronic beam steering and high gain at the cost of increased complexity and implementation cost. Parabola antennas are widely used in satellite ground stations because of their high gain and narrow beamwidth, and they are commonly applied to both LEO and geostationary orbit (GEO) satellite tracking [10]. However, their narrow beamwidth imposes strict pointing accuracy requirements, and even small pointing errors can degrade received signal strength, particularly when tracking fast-moving LEO satellites. Parabola antennas can be configured with different numbers of rotational axes, and conventional two-axis systems typically control azimuth and elevation. Although this structure is simple and cost-effective, tracking satellites near the zenith may cause rapid azimuth changes and gimbal lock, making continuous tracking difficult near an elevation angle of 90° [11,12].

A three-axis parabola antenna mitigates this limitation by introducing an additional mechanical axis, such as a tilt axis, which provides an extra degree of freedom for avoiding tracking discontinuities. However, the additional axis creates multiple possible antenna attitudes for the same pointing direction, requiring the control software to select a feasible attitude while considering axis limits, cable-wrap limits, angular velocity limits, actuator characteristics, and axis coupling. Therefore, in three-axis systems, gimbal lock should be treated not only as a mechanical issue but also as a software-level control problem involving singularity avoidance and coordinated attitude selection. The genericity of a three-axis antenna controller also depends on how antenna-specific mechanical characteristics are separated from the core control logic. If parameters such as axis order, motion range, cable-wrap limits, angular velocity limits, and mechanical offsets are embedded directly in the control logic, the controller becomes tightly coupled to a specific antenna structure; therefore, these properties should be managed as externally configurable specifications.

Antenna tracking methods are generally divided into closed-loop tracking based on received signal feedback and open-loop tracking based on orbital information. Closed-loop tracking can dynamically compensate for pointing errors using real-time signal feedback, but its performance depends on signal quality, signal-to-noise ratio, and initial signal acquisition, and it may also suffer from side-lobe tracking [13]. In contrast, open-loop tracking predicts the satellite position using predefined orbital information such as Two-Line Elements (TLE) and generates antenna pointing commands through orbit propagation and coordinate transformation [14]. Because it can provide deterministic and repeatable tracking without continuous signal feedback, this approach is suitable for automated ground station operation and LEO satellite tracking with rapid motion and short visibility windows. However, open-loop tracking for three-axis parabola antennas requires not only orbit prediction but also transformation of the predicted satellite position into a feasible antenna attitude while avoiding singular configurations and satisfying antenna-specific constraints. Therefore, a software control framework integrating orbit prediction, coordinate transformation, gimbal lock avoidance, and antenna specification reflection is required.

## 3. Related Work

This section analyzes the structures and control methods of antenna control systems presented in previous works and compares them in terms of tracking accuracy and continuity. In particular, it focuses on the limitations of continuous satellite tracking in conventional antenna systems, which arise from issues such as gimbal lock phenomena and dependence on manual initialization operations.

C. Cho et al. [7] developed a satellite tracking system that improves reception performance for specific satellites using the step-tracking method on a two-axis antenna platform. In this method, the antenna is first manually positioned toward a region where the satellite signal can be received, and then it tracks the satellite by moving the antenna in small angular steps around the point of maximum signal level. However, this approach requires manual initialization to move the antenna into the initial reception region, during which tracking loss may occur. Additionally, due to the nulls between the main lobe and side lobes, the antenna may mistakenly align with a side lobe, resulting in inaccurate tracking. The system’s performance also depends on the operator’s experience and proficiency, leading to inconsistent results. Therefore, the step-tracking method has difficulty ensuring tracking accuracy and does not support continuous satellite tracking.

Thejaswini M. N et al. [8] presented an antenna control system for intelligent satellite tracking that calculates the antenna’s azimuth and elevation angles based on TLE data. By utilizing TLE and the SGP4 model, the system predicts the orbit of a specific satellite and generates a tracking schedule, thereby addressing the tracking loss problem that occurs during the scan phase of the conventional step-tracking algorithm. However, since the system lacks a processing mechanism for tracking schedules involving gimbal lock, it cannot ensure continuous satellite tracking. Furthermore, the control logic is tightly coupled to a specific antenna configuration, limiting its reusability across different antenna platforms.

Oguz K. H. et al. [9] employed a method that calculates antenna pointing angles using a TLE-based satellite position prediction algorithm. In addition, the research presented a dynamic solution to the gimbal lock problem by utilizing a four-axis parabola antenna, which adds a polarization axis to the conventional azimuth, elevation, and tilt axes of a three-axis system. However, the work did not present an algorithm capable of generating tracking schedules for satellites that actually experience gimbal lock. Moreover, there was a lack of experimental validation data demonstrating the effectiveness of the presented gimbal lock mitigation approach, which limits its practical applicability. In particular, no large-scale orbital analysis was conducted to verify robustness across diverse gimbal-lock-prone satellite scenarios. Furthermore, the use of a four-axis parabola antenna introduces mechanical and computational complexity, making coordinate calculation and antenna control more difficult [15].

The aforementioned works have improved the accuracy and continuity of satellite tracking by applying TLE-based orbital prediction and multi-axis antenna structures. However, most of these works lack control verification using actual antenna hardware, and the complexity of multi-axis configurations makes system implementation and maintenance difficult. In addition, they show limited real-time handling of gimbal lock situations and insufficient flexibility to accommodate various antenna specifications because antenna-dependent parameters are often embedded in the control logic.

Therefore, there is a need for an efficient control system that can operate reliably in real environments and be adapted to a wide range of antenna configurations. To address these issues, this paper presents a generic three-axis parabola antenna control software that integrates TLE-based orbit prediction, a gimbal lock avoidance algorithm, and an antenna specification reflection function. The proposed system separates antenna-dependent properties, such as axis configuration, motion range, cable-wrap limits, and mechanical constraints, from the core tracking algorithm. This separation enables stable operation in real hardware environments and allows the same tracking logic to be reused for antennas with different sizes, geometries, and operational constraints. Table 1 compares the advantages and limitations of the related works with those of the presented system. In Table 1, accuracy is classified into three levels: Low, Moderate, and High. Continuity and flexibility are evaluated using three categories: Supported, Limited, and Not Supported. In this comparison, Not Supported indicates that the corresponding capability was not demonstrated or validated in the related work.

## 4. NSTracker

This section presents NSTracker, a three-axis parabola antenna control software developed to ensure continuous and stable satellite tracking across diverse orbital and antenna environments. Section 4.1 describes the overall architecture and clarifies the functional relationships among core modules. Section 4.2 presents the configuration and features of the user interface. Section 4.3 describes the core service modules comprising the Tracking Service, Operating Service, and Database. Section 4.4 details the operational algorithms, including solar tracking, satellite tracking, and the gimbal lock avoidance strategy for three-axis control.

### 4.1. System Architecture

**Figure 1:**
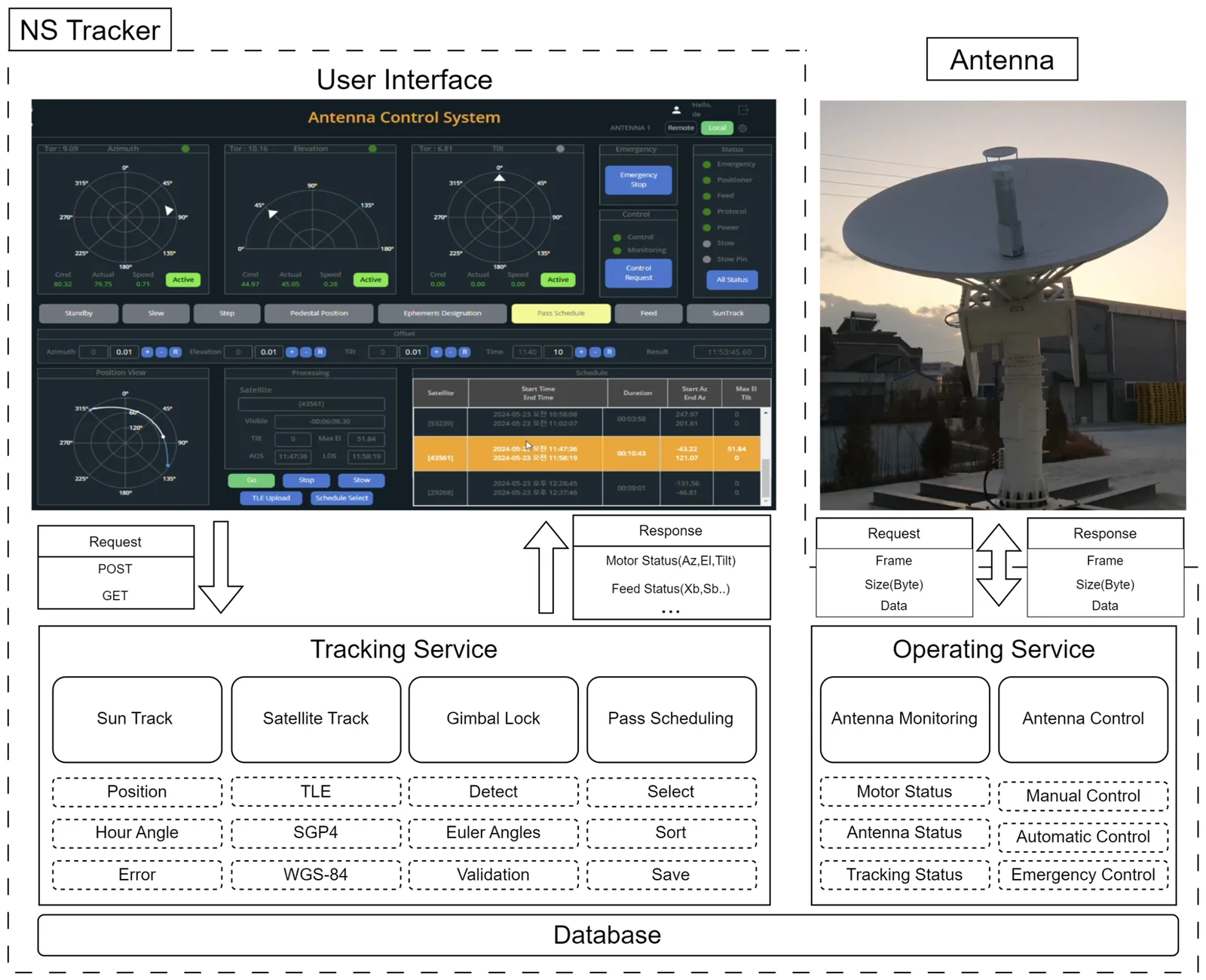
Functional compositionof NSTracker.

The three-axis parabola antenna control software presented in this paper consists of two main components: the NSTracker User Interface (UI) and the NSTracker Service Module, which comprises the Tracking Service, Operating Service, and Database, as shown in Figure 1. The NSTracker UI serves as the primary interface through which users issue control commands and provide configuration inputs to the NSTracker Service Module. When a user enters TLE data, antenna parameters, or tracking mode selections via the UI, these inputs are forwarded to the Service Module, where they are used to generate tracking schedules and drive antenna control. The Tracking Service performs orbit prediction, gimbal lock detection and avoidance, and multi-satellite scheduling; the resulting schedules are persisted in the Database for execution and long-term analysis. The Operating Service references the schedules stored in the Database to drive each antenna axis motor in real time, while continuously monitoring antenna status through sensor feedback. Monitoring data are streamed back to the UI output panel, enabling operators to observe tracking status and system health in real time. The Database stores satellite information, antenna specifications, and ground station parameters, supplying them as inputs to the control algorithms while also managing real-time tracking logs.

A fundamental design principle of NSTracker is the strict separation between the core tracking logic and antenna-specific parameters. Conventional satellite tracking software [7,8,9,15] is typically developed for a specific antenna model, requiring software modification or redevelopment when the antenna hardware or operational environment changes. NSTracker addresses this limitation through the antenna specification reflection function, which automatically supplies antenna-specific parameters—including tilt angle, angular velocity limits, and axis operational ranges—to the tracking algorithms without modifying the core logic. NSTracker addresses this limitation through the antenna specification reflection function. Instead of embedding antenna-dependent parameters into the tracking algorithm, NSTracker manages them as external specifications, including axis configuration, tilt angle, axis operational ranges, angular velocity limits, cable-wrap limits, and mechanical constraints.

During tracking, these specifications are used to evaluate multiple candidate antenna attitudes for the same satellite pointing direction and select a feasible attitude that satisfies the mechanical and operational constraints of the target antenna. As a result, the same orbit prediction, coordinate transformation, gimbal lock avoidance, and scheduling logic can be reused across different three-axis parabola antenna environments without code-level modification. When a new antenna is introduced, NSTracker can be adapted by updating its specification parameters rather than redesigning the core tracking algorithm. This architecture provides the basis for the genericity of the presented software and supports stable operation in real hardware environments.

### 4.2. User Interface

The NSTracker UI is designed with an intuitive interface that allows users to configure antennas, perform manual control, execute solar and satellite tracking, manage multiple satellite schedules, and monitor antenna status in real time. The UI consists of two main panels: the Input & Control Panel and the Output Panel. The Input & Control Panel allows users to input configuration data and execute control commands for antenna tracking, while the Output Panel visually displays antenna status and tracking results. This structure enables both intuitive operation and real-time system monitoring.

The Input & Control Panel provides key configuration and operational commands for antenna control, including TLE data entry, antenna control parameter settings, and tracking mode switching. Its detailed functions include satellite information input, tracking configuration, and control commands.

- Satellite Information Input: Users can load TLE data of the target satellite or manually enter latitude, longitude, and altitude values to define the tracking target. Manual azimuth and elevation inputs are also supported for non-satellite targets.
- Tracking Configuration: Users can select tracking modes (automatic, manual, or scheduled), activate specific control axes, and set parameters such as tracking speed and acceleration limits. This flexibility allows the software to adapt to various antenna specifications.
- Control Commands: Users can execute initialization, start or stop tracking, and perform manual adjustments. All control commands are transmitted to the antenna controller in real time, and the system immediately receives feedback to maintain stable operation.

The Output Panel visually displays the real-time operational status and tracking results of the antenna system. Through this panel, operators can intuitively monitor system health, tracking accuracy, and schedule progress.

- Axis Status Display: Shows the angle, speed, and operation status (Idle/Tracking/Error) of each axis—Elevation, Azimuth, and Polarization—in real time. Status changes are visually distinguished through color codes and warning icons.
- Target Visualization: Displays the positions of the satellite and the Sun based on a sky map coordinate system, enabling comparison with the antenna’s pointing vector to assess tracking errors. This allows operators to evaluate real-time tracking accuracy and correction status at a glance.
- Schedule and Log Information: Provides registered tracking schedules and historical tracking logs in tabular form. The log includes tracking time, signal strength, and error values, which can be utilized for operational analysis and performance evaluation.

### 4.3. Service Module

The NSTracker Service Module consists of three components—Tracking Service, Operating Service, and a Database—to support satellite tracking and antenna operation. The Tracking, Operating, and Database modules operate in close coordination to ensure both high tracking accuracy and stable antenna operation. Through this integrated architecture, the system enables continuous and automated satellite tracking across various satellite environments while simultaneously ensuring reliable antenna control and efficient data management.

The Tracking Service is the core module responsible for orbit prediction and real-time tracking. It ensures the acquisition of high-quality data through precise position calculation and enables continuous and reliable satellite tracking according to the defined schedule. The Tracking Service consists of four modules: Solar Tracking, Satellite Tracking, Keyhole, and Satellite Scheduling.

- Solar Tracking: Predicts the position of the Sun using the ground station’s location as input and generates a solar tracking schedule. This compensates for azimuth offset errors occurring during antenna installation, ensuring high tracking accuracy during satellite operations.
- Satellite Tracking: Calculates the satellite’s position using TLE data and the SGP4 algorithm [16], then generates a tracking schedule after converting the positions to the WGS-84 coordinate system.
- Keyhole: When a gimbal lock condition is detected, computes an Euler-angle-based three-axis rotation schedule to avoid the problem and verifies the feasibility of continuous tracking under that motion sequence.
- Satellite Scheduling: Allows users to select multiple satellite tracking schedules, which are automatically sorted in chronological order and stored as a single integrated schedule, executed autonomously at the designated time.

The Operating Service provides functions for controlling and monitoring the antenna components and axis motors. It is composed of two modules: Antenna Monitoring and Antenna Control.

- Antenna Monitoring: Collects real-time operational data—including motor status, sensor readings, and network connectivity of each axis—and visually displays this information on the user interface.
- Antenna Control: Precisely drives the antenna according to control commands from the system or user, ensuring stable operation and control accuracy during tracking.

The Database stores satellite information, antenna specifications, and ground station parameters, which are used as input data for the control algorithms. It also manages the tracking schedules and real-time tracking logs produced by the satellite tracking algorithms, supporting both stable autonomous control and long-term data analysis. These data can later be used for algorithm performance validation, error correction, and maintenance analysis.

### 4.4. Algorithm

**Figure 2:**
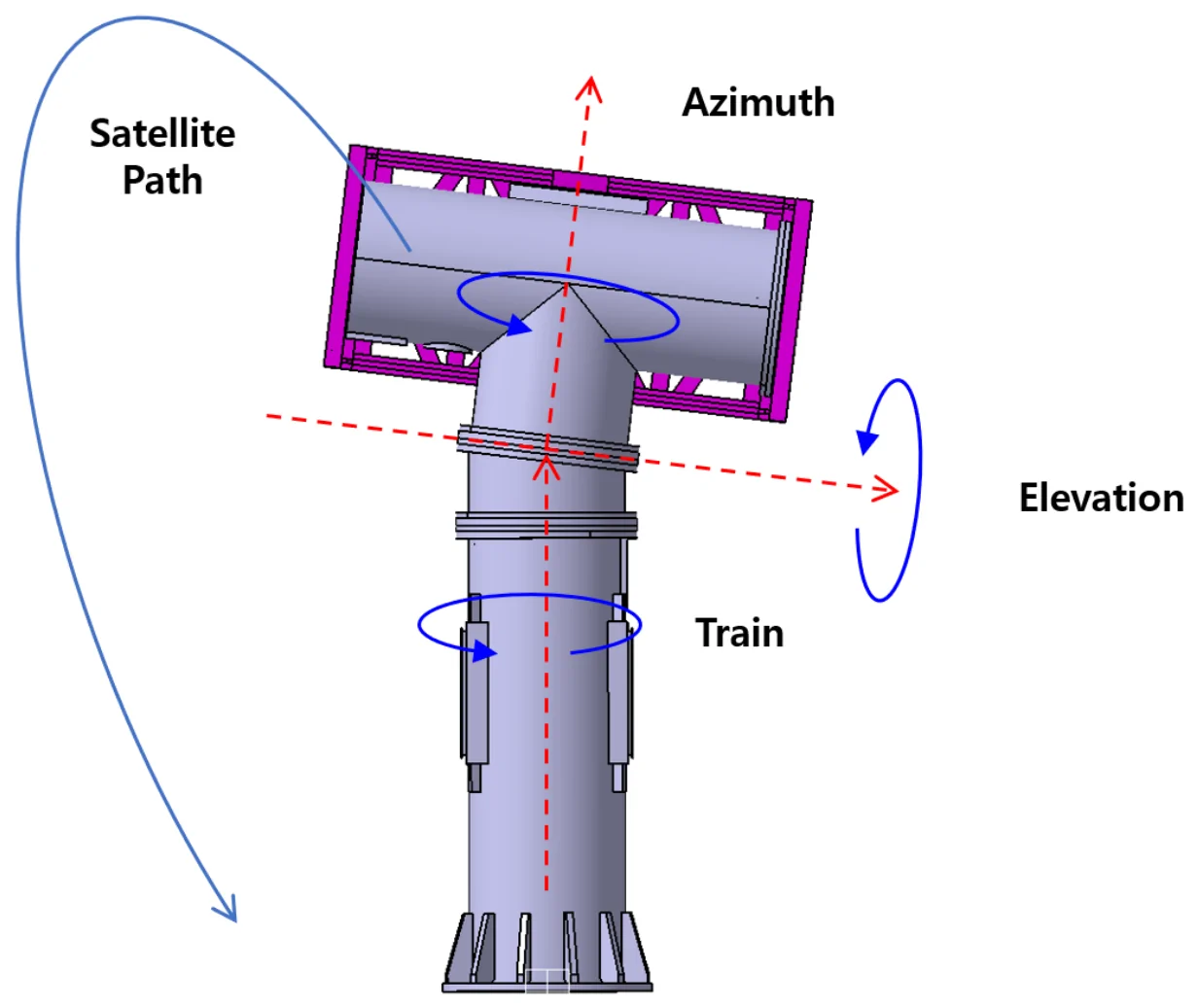
Solution of avoiding gimbal lock problem.

**Figure 3:**
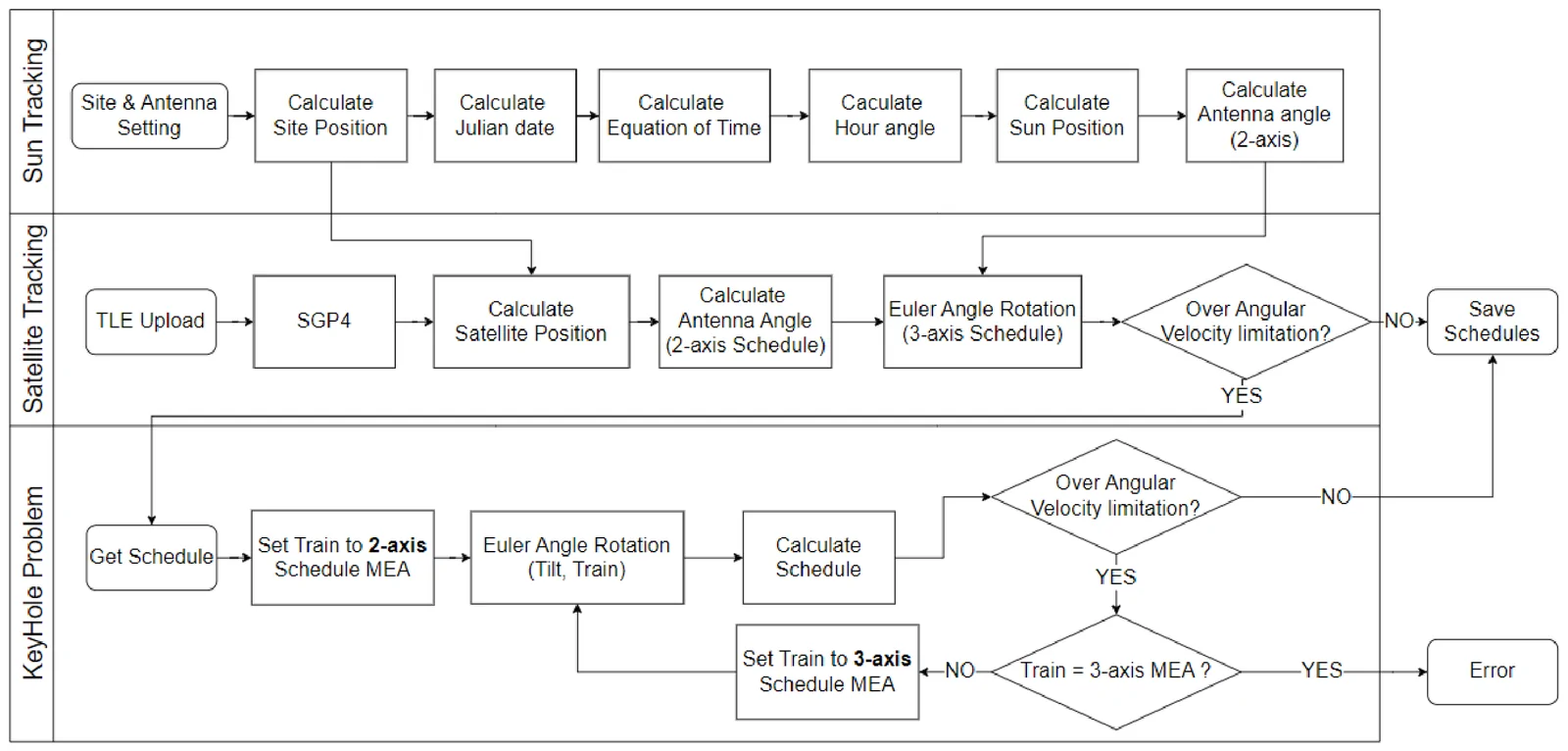
Sequence diagram of tracking algorithm.

A satellite tracking system must continuously maintain data communication links by accurately calculating the position of fast-moving satellites from the observation point (ground station). To achieve precise tracking, solar tracking is first performed to compensate for mechanical and installation errors—including azimuth misalignment between true north and magnetic north—thus reducing structural deviations before applying the satellite tracking algorithm. The satellite tracking algorithm computes the real-time position of the satellite, enabling the antenna to accurately follow its trajectory. In addition, to ensure uninterrupted satellite communication, the gimbal lock phenomenon must be avoided. Gimbal lock avoidance is achievable in three-axis antenna systems. In this paper, the Train axis—the base rotation axis of the three-axis antenna mount, positioned below the Azimuth and Elevation axes as shown in Figure 2—is utilized to track satellites that cannot be followed by conventional two-axis systems. The antenna-specific parameters used across the solar tracking, satellite tracking, and gimbal lock avoidance algorithms—including tilt angle, angular velocity limits, and axis operational ranges—are automatically supplied through the antenna specification reflection function, decoupled from the core tracking logic, enabling universal applicability across diverse antenna environments. The overall flow of the satellite tracking software is shown in Figure 3.

Solar tracking is essential not only for tracking the Sun but also for correcting azimuth deviations between true north and magnetic north that may occur during antenna installation [17]. For solar tracking, the required inputs are the ground station’s position, date and time, operational angle of each antenna axis, angular velocity limits, and the tilt-axis inclination. The time information is converted to Julian Date and refined using the Equation of Time and Hour Angle for precise temporal correction. Based on the calculated time data, the LLH (Latitude, Longitude, Height) coordinates of the Sun are determined. Using the derived solar position, a two-axis antenna schedule (azimuth and elevation) is first generated for each time step.

Finally, the antenna pointing direction determined by the two-axis antenna angles is represented as a Cartesian vector and transformed into the corresponding three-dimensional coordinate frame using an Euler-angle-based rotation matrix, as expressed in Equation (1) [18].

(1) $$p_{r}=Rp_{o},$$

where $p_{o}={\left[x_{o},y_{o},z_{o}\right]}^{T}\in R^{3}$ denotes the original pointing vector, and $p_{r}={\left[x_{r},y_{r},z_{r}\right]}^{T}\in R^{3}$ denotes the rotated pointing vector. $R\in R^{3\times 3}$ denotes the rotation matrix constructed from the Euler angles ϕ, θ, and ψ, representing rotations about the *x*-, *y*-, and *z*-axes, respectively.

Satellite tracking is the function that calculates the satellite’s position and generates a schedule by converting it into corresponding antenna angles. The Two-Line Element (TLE) is an international standard format that represents a satellite’s orbit using two lines of data, which, when combined with the SGP4 algorithm, enables the calculation of a satellite’s position at a specific time. In this paper, TLE data are utilized to ensure both the accuracy and efficiency of orbital prediction.

The SGP4 algorithm propagates the orbital elements contained in the TLE as a function of the elapsed time from the TLE epoch and calculates the satellite position and velocity vectors in the True Equator, Mean Equinox (TEME) coordinate system at each time step [19].

(2) $$\left[r_{\mathrm{TEME}}\left(t\right),v_{\mathrm{TEME}}\left(t\right)\right]=\mathrm{SGP4}\left(E_{\mathrm{TLE}},\Delta t\right),\;\Delta t=t-t_{\mathrm{epoch}},$$

Here, $E_{\mathrm{TLE}}$ denotes the set of orbital parameters contained in the TLE, including the mean motion, eccentricity, inclination, right ascension of the ascending node, argument of perigee, mean anomaly, and BSTAR drag term. The vectors $r_{\mathrm{TEME}}\left(t\right)={\left[x_{t},y_{t},z_{t}\right]}^{T}$ and $v_{\mathrm{TEME}}\left(t\right)={\left[v_{x,t},v_{y,t},v_{z,t}\right]}^{T}$ denote the satellite position and velocity vectors in the TEME coordinate system at time *t*, respectively.

The TEME position vector generated by SGP4 is subsequently transformed into an Earth-fixed coordinate system and, when required, converted into latitude, longitude, and height coordinates referenced to the WGS-84 ellipsoid [16]. Using the WGS-84 ellipsoid, the geodetic latitude $\phi_{g}$, longitude $\lambda_{g}$, and height $h_{g}$ of the ground station are converted into ECEF coordinates as follows.

(3) $$\begin{array}{cc} N(\phi_{g}) & =\frac{a}{\sqrt{1-e^{2}\sin^{2}\phi_{g}}},\\ X_{g} & =\left(N\left(\phi_{g}\right)+h_{g}\right)\cos\phi_{g}\cos\lambda_{g},\\ Y_{g} & =\left(N\left(\phi_{g}\right)+h_{g}\right)\cos\phi_{g}\sin\lambda_{g},\\ Z_{g} & =\left[N\left(\phi_{g}\right)\left(1-e^{2}\right)+h_{g}\right]\sin\phi_{g}, \end{array}$$

Here, *a* denotes the semi-major axis of the WGS-84 ellipsoid, *e* denotes its first eccentricity, and $N(\phi_{g})$ denotes the prime vertical radius of curvature. In the Earth-fixed coordinate system, the relative position vector from the ground station to the satellite is calculated as

(4) $$\Delta r_{\mathrm{ECEF}}=r_{s,\mathrm{ECEF}}-r_{g,\mathrm{ECEF}}=\left[\begin{array}{c} \Delta X\\ \Delta Y\\ \Delta Z \end{array}\right],$$

The relative ECEF vector is then rotated into the local East–North–Up (ENU) coordinate system using the ground-station latitude $\phi_{g}$ and longitude $\lambda_{g}$ as

(5) $$\left[\begin{array}{c} E\\ N\\ U \end{array}\right]=\left[\begin{array}{ccc} -\sin\lambda_{g} & \cos\lambda_{g} & 0\\ -\sin\phi_{g}\cos\lambda_{g} & -\sin\phi_{g}\sin\lambda_{g} & \cos\phi_{g}\\ \cos\phi_{g}\cos\lambda_{g} & \cos\phi_{g}\sin\lambda_{g} & \sin\phi_{g} \end{array}\right]\left[\begin{array}{c} \Delta X\\ \Delta Y\\ \Delta Z \end{array}\right].$$

Here, $r_{s,\mathrm{ECEF}}$ and $r_{g,\mathrm{ECEF}}$ denote the ECEF position vectors of the satellite and ground station, respectively, while ΔX, ΔY, and ΔZ denote their relative Cartesian components. The variables *E*, *N*, and *U* represent the East, North, and Up components of the satellite pointing vector in the local coordinate frame centered at the ground station.

The ENU pointing vector is then transformed according to the fixed antenna tilt angle α using the Euler-angle-based rotation matrix shown in Equation (6) [18]. The system checks whether the angular velocity of the schedule exceeds the defined velocity limits. If it does not exceed the limit, the schedule is stored; otherwise, the process transitions to the gimbal lock avoidance algorithm.

(6) $$\left[\begin{array}{c} E_{r}\\ N_{r}\\ U_{r} \end{array}\right]=\left[\begin{array}{ccc} \cos\alpha & 0 & \sin\alpha \\ 0 & 1 & 0\\ -\sin\alpha & 0 & \cos\alpha \end{array}\right]\left[\begin{array}{c} E\\ N\\ U \end{array}\right]$$

where *E*, *N*, and *U* denote the East, North, and Up components of the satellite pointing vector in the local ENU coordinate system, respectively. The angle α denotes the fixed tilt angle of the antenna determined by its mechanical configuration. The variables $E_{r}$, $N_{r}$, and $U_{r}$ represent the corresponding vector components after applying the tilt rotation. The resulting ENU pointing vector is subsequently converted into the antenna Azimuth and Elevation pointing angles using the coordinate transformation and angle-calculation equations defined in this section.

**Figure 4:**
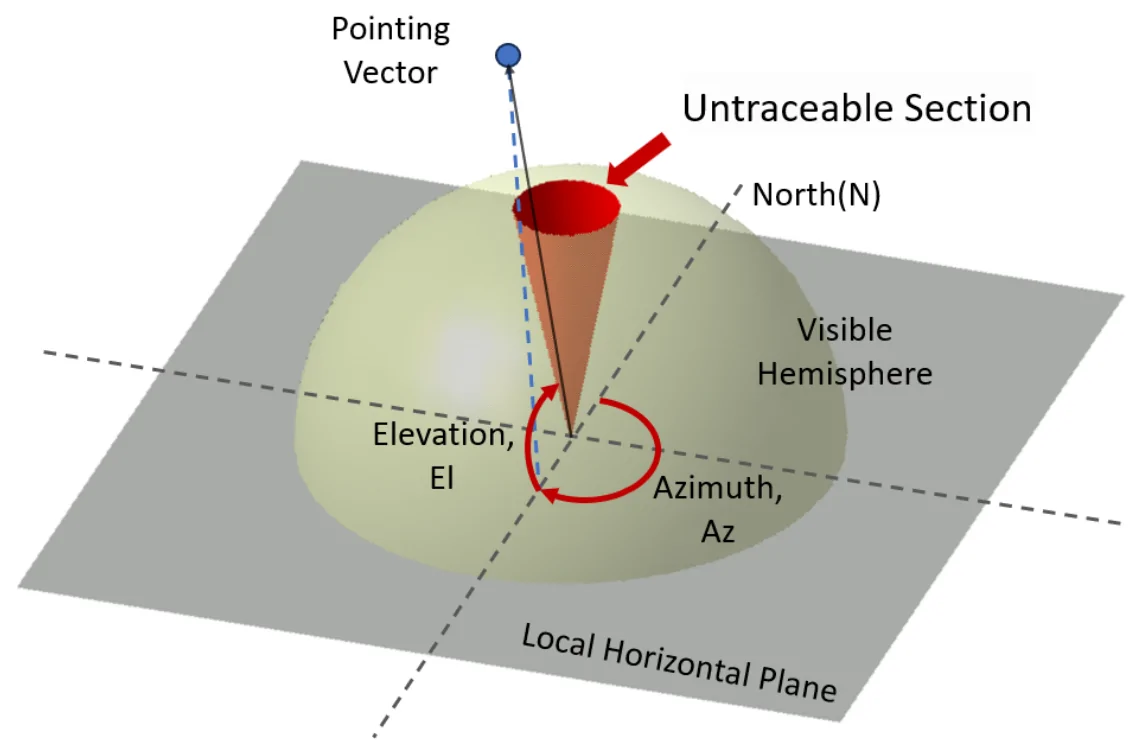
Geometric representation of the gimbal lock problem in a two-axis antenna. The gray plane denotes the local horizontal plane, the shaded hemisphere denotes the antenna tracking space, and the red cone near the zenith represents the keyhole (untraceable) region. The azimuth angle (Az) is measured on the horizontal plane, and the elevation angle (El) is measured upward from the horizontal plane to the pointing vector.

The gimbal lock problem, as illustrated in Figure 4, occurs when the maximum elevation angle of a tracking schedule approaches $90^{\circ }$, causing the required azimuth angular velocity to become unbounded. The gray plane represents the local horizontal plane, and the hemisphere represents the antenna tracking space. Azimuth (Az) is measured on the horizontal plane, while elevation (El) is measured upward from the horizontal plane. The red cone around the zenith ($El\approx 90^{\circ }$) indicates the keyhole region in which the required azimuth angular velocity can exceed the antenna’s mechanical limit. This results in a region where tracking becomes impossible, leading to discontinuities in satellite tracking [5].

To avoid this problem, the Keyhole module applies a gimbal lock avoidance algorithm using the Train axis rotation when the angular velocity of the generated two-axis tracking schedule exceeds the mechanical angular velocity limit of the antenna. In this paper, the antenna is tilted $7^{\circ }$ toward the East, as shown in Figure 2, and the Train angle β is therefore determined so that the antenna can observe the satellite at its maximum elevation from an oblique orientation. The azimuth corresponding to the maximum elevation point of the two-axis tracking schedule is defined as the maximum-elevation azimuth (MEA). The Train angle β is then determined from the MEA using the piecewise transformation defined in Equation (7).

(7) $$\beta =\left\{\begin{array}{cc} \mathrm{MEA}+90^{\circ }, & 0^{\circ }\leq \mathrm{MEA}<180^{\circ },\\ \mathrm{MEA}-270^{\circ }, & 180^{\circ }\leq \mathrm{MEA}<360^{\circ }. \end{array}\right.$$

Here, β denotes the Train-axis angle, and the angular quantities in Equation (7) are expressed in degrees. The MEA denotes the azimuth corresponding to the maximum elevation point of the two-axis tracking schedule, and its domain is defined as $0^{\circ }\leq \mathrm{MEA}<360^{\circ }$. The three-axis tracking schedule is then recalculated by applying the fixed tilt angle α and the Train-axis angle β to the ENU pointing vector, as defined in Equation (8).

(8) $$\left[\begin{array}{c} E_{r}\\ N_{r}\\ U_{r} \end{array}\right]=\left[\begin{array}{ccc} \cos\alpha & 0 & \sin\alpha \\ 0 & 1 & 0\\ -\sin\alpha & 0 & \cos\alpha \end{array}\right]\left[\begin{array}{ccc} \cos\beta & -\sin\beta & 0\\ \sin\beta & \cos\beta & 0\\ 0 & 0 & 1 \end{array}\right]\left[\begin{array}{c} E\\ N\\ U \end{array}\right]$$

Here, α denotes the fixed tilt angle determined by the mechanical configuration of the antenna, and β denotes the Train-axis angle determined from the MEA according to Equation (7). The angular velocities of the transformed three-axis tracking schedule are recalculated and compared with the predefined mechanical angular velocity limits of the antenna. For each antenna axis *i*, the angular velocity between two consecutive schedule points is calculated as defined in Equation (9).

(9) $$\omega_{i}\left(t_{k}\right)=\frac{\theta_{i}\left(t_{k}\right)-\theta_{i}\left(t_{k-1}\right)}{t_{k}-t_{k-1}},\;\left|\omega_{i}\left(t_{k}\right)\right|\leq \omega_{i,\max},$$

where $\theta_{i}\left(t_{k}\right)$ denotes the commanded angle of antenna axis *i* at time $t_{k}$, $\omega_{i}\left(t_{k}\right)$ denotes the corresponding angular velocity, and $\omega_{i,\max}$ denotes the mechanical angular velocity limit of axis *i*. The index *i* represents the Azimuth, Elevation, or Train axis, and $t_{k}$ and $t_{k-1}$ denote two consecutive schedule time points. If the angular velocities of all three axes satisfy their corresponding limits, the transformed schedule is stored for execution; otherwise, an error flag is generated.

The variables *E*, *N*, and *U* denote the components of the satellite pointing vector in the local ENU coordinate system, whereas $E_{r}$, $N_{r}$, and $U_{r}$ denote the corresponding components after applying the Train and tilt rotations. In this study, the azimuth angle is defined with North as the $0^{\circ }$ reference direction, and the calculated azimuth is normalized according to the operational range of the antenna. In Equation (8), the rotation associated with the Train angle β is applied first, followed by the rotation associated with the fixed tilt angle α. The Azimuth and Elevation command angles of the three-axis antenna are calculated from the rotated ENU pointing vector $\left(E_{r},N_{r},U_{r}\right)$ as follows.

(10) $$Az_{3}=\mathrm{atan}2\left(E_{r},N_{r}\right)$$

(11) $$El_{3}=\mathrm{atan}2\left(U_{r},\sqrt{E_{r}^{2}+N_{r}^{2}}\right)$$

Here, $Az_{3}$ and $El_{3}$ denote the Azimuth and Elevation command angles of the three-axis antenna after the coordinate transformation, respectively. The Train-axis command angle is given by β, which is determined from the MEA according to Equation (7).

## 5. Experiment and Analysis

### 5.1. Environment

**Figure 5:**
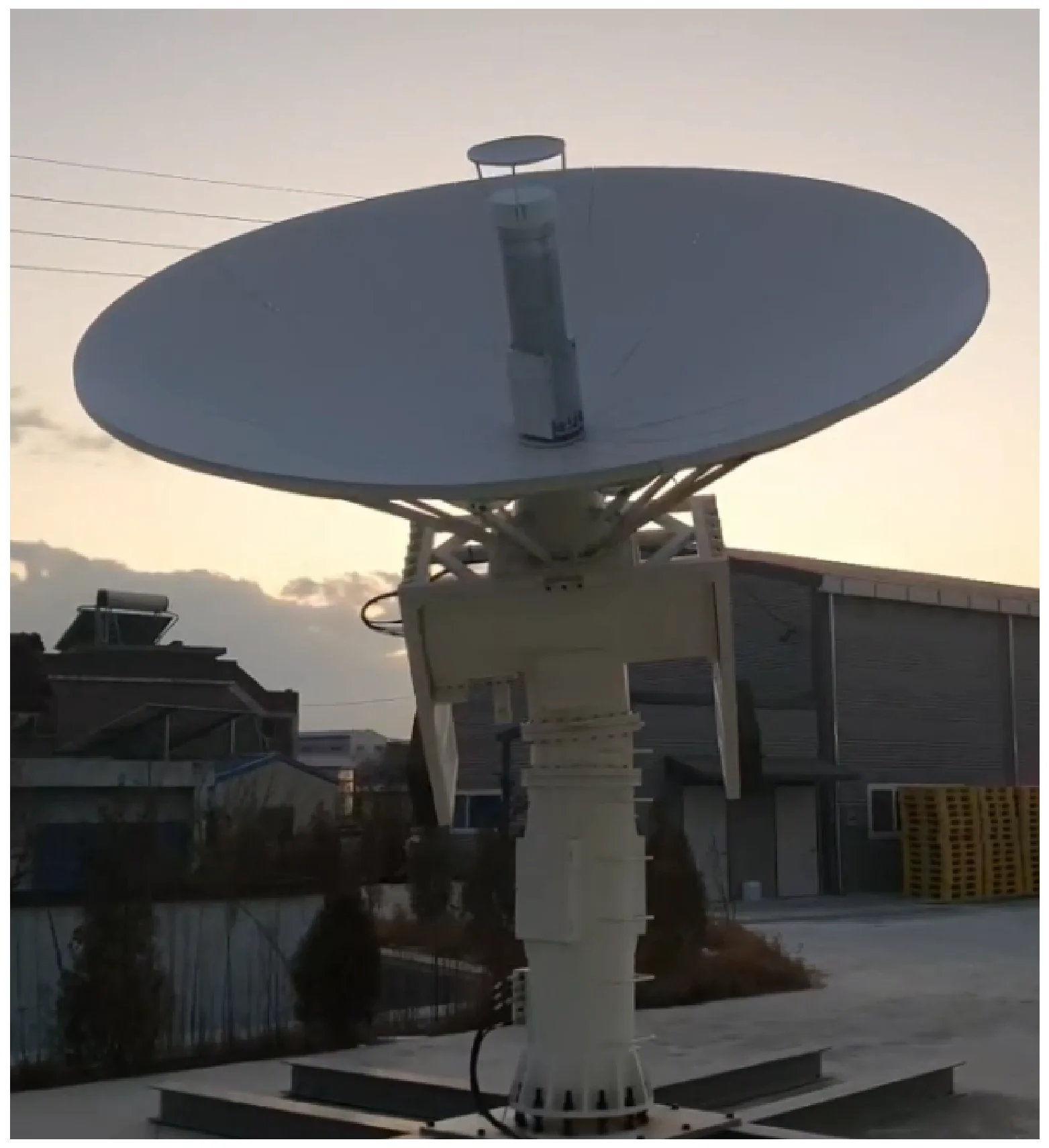
Experiment antenna.

The Ground-to-Tilt, Tilt-to-Azimuth, and Azimuth-to-Elevation parameters in Table 2 represent the physical distances between the corresponding rotational hinge axes in the antenna structure. The antenna used in the experiment is a three-axis parabola antenna (RV-423, GTL Co., Ltd., Changwon, Republic of Korea) with a tilt axis inclined $7^{\circ }$ toward the East, as shown in Figure 5, and its detailed specifications are listed in Table 2. The experimental site is located at a latitude of $35.31754^{\circ }$, longitude of $128.60851^{\circ }$, and an altitude of 12 m above sea level. For the experiment, TLE data provided by CelesTrak were used as the source of satellite orbital information [20].

### 5.2. Solar Tracking

The purpose of the solar tracking experiment is to verify whether the antenna feed accurately points toward the Sun when the solar tracking function is activated and operates based on the calculated solar schedule. During the experiment, the system must maintain a constant signal gain level without applying any offset correction. The experiment consists of three stages:

- Calibration Stage: Previous research has demonstrated that antenna installation and mechanical or structural factors can introduce pointing errors, and reported that pointing calibration of a three-axis antenna reduced the error from approximately $3.5^{\circ }$ before calibration to within $0.5^{\circ }$ after calibration [21]. Therefore, in the calibration stage of this study, the reference pointing direction is obtained from the calculated solar tracking schedule, and the Train-axis angle is adjusted to identify the angle that maximizes the received signal strength, thereby compensating for the azimuth deviation caused by antenna installation errors.
  The optimal Train-axis correction angle is defined as
  (12) $$\beta^{*}=\underset{\beta \in B}{\mathrm{arg max}}\;G\left(\beta \right),$$
  where β denotes the Train-axis angle, B denotes the range of Train angles searched during calibration, G(β) denotes the received signal strength measured at angle β, and $\beta^{*}$ represents the correction angle corresponding to the maximum received signal strength.
- Continuous Tracking Stage: In the continuous tracking stage, the system updates the current time at one-second intervals from sunrise to sunset and calculates the solar position at each time step to generate the corresponding antenna pointing angles. At each time step,
  (13) $$t_{k}=t_{0}+k\Delta t,\;\Delta t=1\;s.$$
  At each time step, the calculated solar pointing vector is converted into the Azimuth, Elevation, and Train command angles using the coordinate transformation procedure defined in Section 4.4. The generated command angles are transmitted to the antenna controller and updated with the newly calculated commands at the next time step. To evaluate the tracking accuracy, the Azimuth and Elevation angles under automatic tracking were recorded at each measurement interval, and the Manual Control function was used to identify the pointing angles corresponding to the maximum received signal strength. The Azimuth and Elevation tracking errors were defined as
  (14) $$e_{Az}=Az_{\mathrm{auto}}-Az_{\mathrm{peak}},\;e_{El}=El_{\mathrm{auto}}-El_{\mathrm{peak}}.$$
  where $Az_{\mathrm{auto}}$ and $El_{\mathrm{auto}}$ denote the pointing angles during automatic tracking, and $Az_{\mathrm{peak}}$ and $El_{\mathrm{peak}}$ denote the reference angles corresponding to the maximum received signal strength.
  The antenna control command generated at time $t_{k}$ is defined by the command vector
  (15) $$\theta_{\mathrm{cmd}}\left(t_{k}\right)=\left[\begin{array}{c} \theta_{Az,\mathrm{cmd}}\left(t_{k}\right)\\ \theta_{El,\mathrm{cmd}}\left(t_{k}\right)\\ \theta_{Tr,\mathrm{cmd}}\left(t_{k}\right) \end{array}\right]=\left[\begin{array}{c} Az(t_{k})\\ El(t_{k})\\ \beta (t_{k}) \end{array}\right].$$
  Here, $\theta_{Az,\mathrm{cmd}}$, $\theta_{El,\mathrm{cmd}}$, and $\theta_{Tr,\mathrm{cmd}}$ denote the target angular position commands transmitted to the Azimuth, Elevation, and Train axes, respectively. At each time step, the newly calculated pointing angles update the target position commands of the corresponding antenna axes, and the antenna controller drives each axis according to these commands.
- Time Stage: To minimize timing errors, real-time GPS-based time information was synchronized with the server time using a GPS-NTP(Network Time Protocol) server. As a result of the synchronization, real-time accuracy of up to 1 ms was achieved [22].

The calibration procedure corrected an initial pointing offset of $18^{\circ }$ by applying the Train-axis correction determined from the maximum received signal strength. To evaluate the accuracy of continuous solar tracking, the Azimuth and Elevation angles under automatic tracking and the received signal strength were recorded at 30-min intervals. At each measurement point, the Azimuth and Elevation angles were finely adjusted to identify the pointing direction corresponding to the maximum received signal strength, which was defined as the reference pointing direction. The differences between the automatically calculated pointing angles and the reference angles corresponding to the maximum received signal strength were defined as the Azimuth and Elevation tracking errors, respectively.

(16) $$|e_{Az}\left(t_{k}\right)|\leq \epsilon_{Az},\;\left|e_{El}\left(t_{k}\right)\right|\leq \epsilon_{El},$$

where $\epsilon_{Az}$ and $\epsilon_{El}$ denote the allowable Azimuth and Elevation pointing errors, respectively, determined according to the pointing characteristics of the antenna. A measurement point is considered to satisfy the tracking-accuracy criterion when both tracking errors remain within their allowable ranges. If either error exceeds its allowable range, the measurement point is recorded as a tracking error and is further examined to determine whether additional alignment or recalibration is required. Table 3 presents the results of a full-day solar tracking experiment performed without manual intervention. The data collected at 30-min intervals showed a maximum angular error of 0.3° relative to the peak gain. In addition, the average signal gain and average maximum gain differed by only 0.13 dB, demonstrating that the antenna maintained stable tracking performance based on RSSI(Received Signal Strength Indicator) measurements [23]. These results verify the accuracy and reliability of the automatic solar tracking function without the need for manual control. Considering that the 3 dB beamwidth of typical parabolic antennas ranges from 0.3° to 1.5°, a maximum angular error of 0.3° falls within operationally acceptable limits, confirming that the solar tracking function achieves sufficient accuracy for practical satellite communication without manual intervention.

### 5.3. Geostationary Satellite Tracking

A geostationary satellite is a satellite that orbits the Earth with the same rotational period as the Earth itself, allowing it to remain fixed over a specific point on the Earth’s surface. The purpose of the geostationary satellite tracking experiment is to verify the reliability of the conversion from two-axis to three-axis antenna angles, which is performed using the SGP4 algorithm and coordinate transformation formulas, by leveraging the stationary characteristics of geostationary satellites. Additionally, the experiment evaluates the accuracy of the automatic tracking system by comparing the signal gain obtained from two-axis and three-axis antenna angles. The experiment proceeds as follows:

First, the TLE data of the target geostationary satellite are used as input to the SGP4 algorithm to calculate the satellite position. The calculated satellite position and the ground-station position are then used to obtain the local ENU pointing vector through the coordinate transformation procedure defined in Section 4.4. The Azimuth and Elevation pointing angles of the two-axis antenna are calculated from the ENU pointing vector, and the received satellite signal strength is measured at the resulting pointing direction. Next, the pointing vector of the three-axis antenna is calculated using the Euler rotation transformation with the tilt angle α and Train angle β defined in Section 4.4. The final Azimuth and Elevation command angles are derived from the rotated ENU vector and applied to the three-axis antenna together with the Train angle β, after which the received signal strength is measured again. The three-axis coordinate transformation is considered to maintain acceptable pointing performance when the absolute difference between the average received signal strengths of the two pointing configurations does not exceed the predefined tolerance $\Delta G_{\max}$.

(17) $$\left|{\bar{G}}_{3\mathrm{axis}}-G_{2\mathrm{axis}}\right|\leq \Delta G_{\max},\;\Delta G_{\max}=1\;\mathrm{dB}.$$

**Figure 6:**
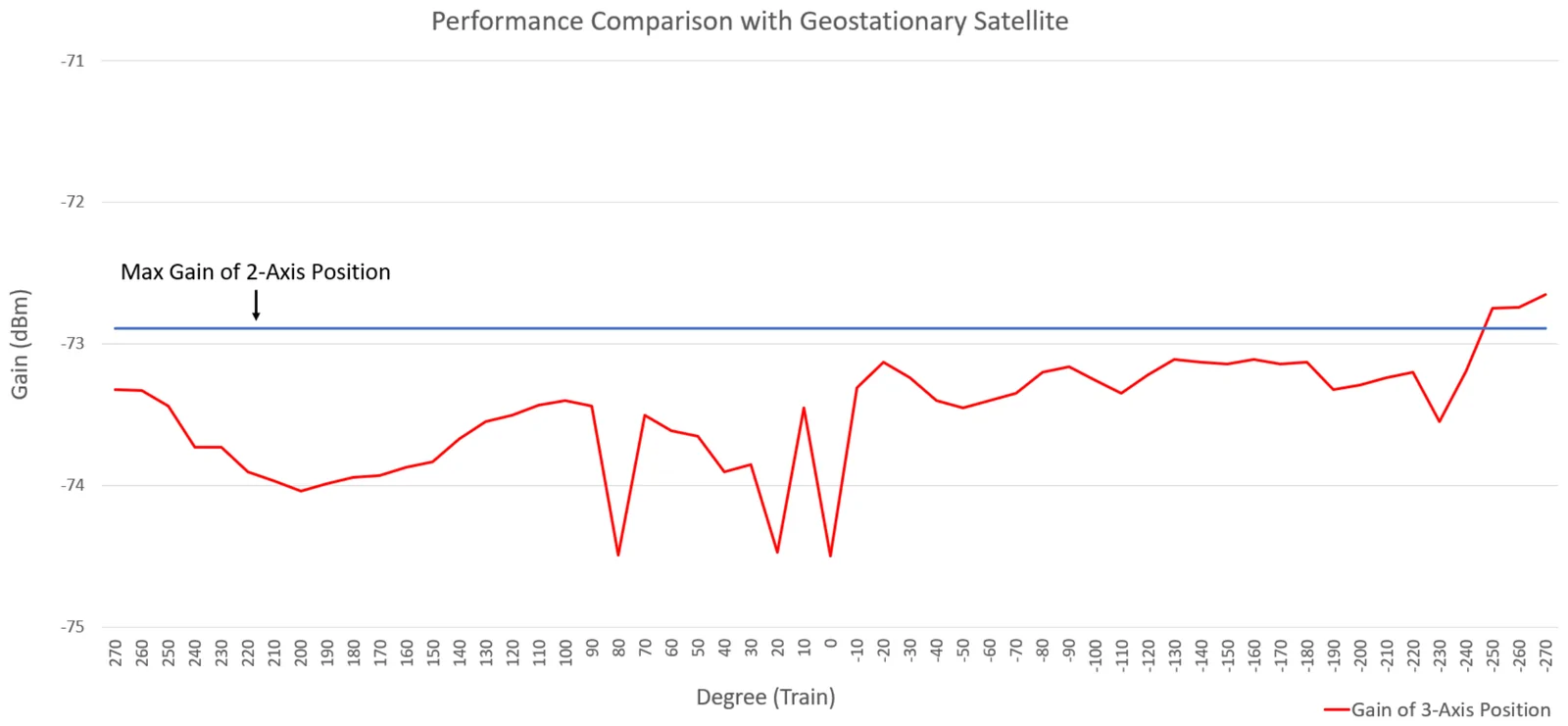
Performance comparison with geostationary satellite.

The target satellite for this experiment is GEO-KOMPSAT-2A, developed by the Korea Aerospace Research Institute (KARI) for meteorological observation and space weather monitoring [24]. The TLE data provided by CelesTrak on 6 March 2024 were used for this experiment, as shown in Table 4. Based on this data, the SGP4 algorithm and coordinate transformations calculated the two-axis antenna angles as Azimuth 198.07° and Elevation 48.61°, and the measured satellite signal gain was –72.89 dBm. The experimental results, illustrated in Figure 6, show the recorded signal gain when the two-axis antenna angles were converted into three-axis antenna angles using a tilt axis inclination of 7° and a train rotation range from 270° to –270°. Across 57 measurements, the average received signal strength of the three-axis antenna was −73.483 dBm, and the absolute difference from the two-axis antenna measurement of −72.89 dBm was 0.593 dB. Since this value satisfies $\left|\Delta G\right|=0.593\;\mathrm{dB}<\Delta G_{\max}=1\;\mathrm{dB}$, the average received-signal variation introduced by the three-axis coordinate transformation was considered to be within the predefined tolerance. Furthermore, all 57 measurements obtained over the full Train rotation range satisfied the criterion $\Delta G_{\max}=1\;\mathrm{dB}$, indicating that the received-signal performance after coordinate transformation remained consistent over the tested Train orientations. This minor deviation is likely caused by external factors such as weather conditions and radio interference during signal transmission.

### 5.4. Low Earth Orbit Satellite Tracking

Low Earth orbit (LEO) satellites orbit the Earth at lower altitudes and higher speeds, allowing a single satellite to have multiple tracking schedules per day. Considering this characteristic, a satellite tracking system must provide the capability to predict satellite paths and schedule tracking operations accordingly. Each satellite tracking schedule must control the antenna’s pointing angles precisely at each time step to maintain a stable signal strength. The purpose of the LEO satellite tracking experiment is to verify the system’s ability to predict and schedule satellite trajectories based on multiple TLE datasets and to evaluate its performance in maintaining consistent signal strength and waveform stability during tracking. The experiment consists of two parts: one for verifying the multi-satellite scheduling function and the other for validating the LEO satellite tracking performance. For the scheduling verification, multiple satellites TLE data are input into the system to compute all tracking schedules expected to occur within a day. The selected schedules are then uploaded to the server to confirm whether the system can automatically track the satellites according to the scheduled times. Next, using the ARIRANG-5 satellite’s tracking schedule, the LEO tracking performance is tested by checking whether the signal strength and waveform remain consistent throughout the tracking session. ARIRANG-5 is a low-Earth-orbit Earth-observation satellite developed by the Korea Aerospace Research Institute (KARI) and is equipped with a Synthetic Aperture Radar (SAR) for all-weather, day-and-night Earth observation [25]. The TLE data for ARIRANG-5 are provided by CelesTrak, as shown in Table 5.

The experimental results are presented in Table 6, which summarizes the signal strength and waveform stability measured over a three-day tracking period of ARIRANG-5. During the tracking process, the system controlled the antenna at 0.1-s intervals based on the predefined tracking schedule, successfully maintaining stable signal strength and waveform continuity from the start to the end of each pass. These results confirm that the multi-satellite scheduling and LEO tracking functions operate reliably, maintaining consistent signal quality throughout each pass without dropout. Given that LEO satellites traverse the sky in under 15 min with rapidly changing angles, achieving zero dropout across all three passes—including one with a maximum elevation of 85.24°—demonstrates that the scheduling and control functions perform robustly even under demanding dynamic conditions.

### 5.5. Gimbal Lock Avoidance

The gimbal lock problem occurs when the maximum elevation angle of a tracking schedule approaches 90 degrees, causing the azimuth angular velocity to increase infinitely and resulting in untrackable schedules. To address this problem, the antenna’s tilt inclination and train rotation are utilized. The purpose of the gimbal lock avoidance experiment is to verify whether satellites experiencing gimbal lock can be continuously tracked by applying train rotation. Since this experiment involves angular velocities that exceed the physical capabilities of the actual antenna hardware, it is conducted in a simulation environment to ensure safety and equipment protection.

**Figure 7:**
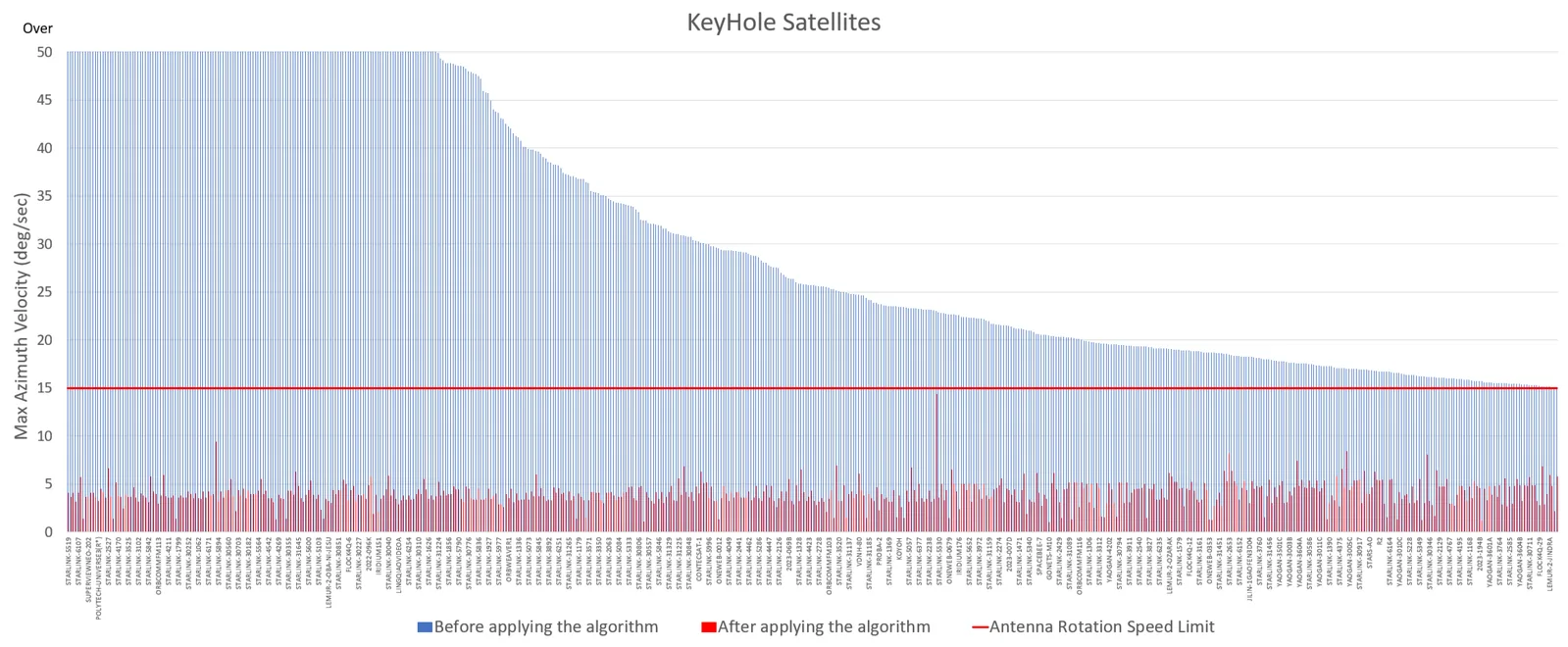
Gimbal Lock Simulation Experiment at 2 May 2024.

The experiment proceeds as follows. All satellite data provided by CelesTrak are analyzed to detect satellites prone to gimbal lock, and their corresponding azimuth angular velocities are measured. After applying the gimbal lock avoidance algorithm, the system measures the azimuth angular velocity again following train rotation to determine whether tracking can be performed within the antenna’s velocity limits. Figure 7 presents the results of the simulation experiment conducted on 2 May 2024. For 597 satellites that experienced gimbal lock, the maximum azimuth angular velocity at their maximum elevation angles exceeded the antenna’s velocity limit of 15°/s. After applying the gimbal lock avoidance algorithm, the maximum azimuth angular velocities for all these satellites were reduced to within the antenna’s velocity limit, confirming that continuous tracking was achievable.

Table 7 summarizes the results of an 18-day simulation experiment. Out of 177,152 satellites analyzed, 11,469 satellites were identified as being affected by gimbal lock. By applying the presented algorithm, all satellites were successfully tracked within the antenna’s maximum angular velocity limit of 15°/s, and no untrackable schedules were observed. Through this experiment, it was verified that the introduction of the gimbal lock avoidance algorithm effectively reduced the azimuth angular velocity of all gimbal lock satellites to within the physical limits of the antenna, enabling stable and reliable satellite tracking. This result represents a significant advancement in solving the tracking challenges near 90° elevation, greatly enhancing the performance and efficiency of the satellite tracking system. All 1469 gimbal lock satellites had azimuth angular velocities that far exceeded the antenna’s 15°/s limit in the two-axis case. The fact that all of them were brought within that limit after applying train rotation confirms that the algorithm provides a universally applicable solution regardless of the severity of the gimbal lock condition.

### 5.6. Antenna Specification

Antenna hardware specifications—such as tilt angle and axis operational ranges—directly affect the accuracy of computed pointing angles; failing to incorporate them into the tracking algorithm results in persistent angular offsets that degrade tracking quality. To quantify these effects and validate the antenna specification reflection function, tracking experiments were conducted on a geostationary satellite with and without incorporating antenna-specific parameters into the algorithm.

In Table 8, Spec O denotes the condition in which the antenna-specific hardware parameters are incorporated into the tracking algorithm, whereas Spec X denotes the condition in which these parameters are not incorporated. When a tracking experiment was conducted on a geostationary satellite without applying hardware specifications, a consistent elevation error of approximately $0.2^{\circ }$ was observed. Subsequently, when the 4 m antenna configuration data were applied under the same conditions, the elevation error was eliminated, confirming that tracking was performed without any offset. Furthermore, similar results were obtained with the 7 m antenna, where tracking was also achieved without offset. These results demonstrate that the antenna specification reflection function reliably compensates for hardware-induced pointing errors, providing consistent tracking performance across antennas of different sizes and configurations. The elimination of pointing offset across both 4 m and 7 m antennas under identical algorithmic conditions confirms that the antenna specification reflection function generalizes effectively across different hardware configurations, without requiring modification to the core tracking logic.

## 6. Conclusions

This paper implemented and validated a three-axis parabola antenna control software designed for accurate and stable satellite tracking in the New Space era. The presented software integrates TLE-based orbital prediction, a gimbal lock avoidance algorithm, and an antenna specification reflection function, ensuring high levels of accuracy, continuity, and generality. Experimental validation using an actual antenna demonstrated that, for the geostationary satellite GEO-KOMPSAT-2A, the presented software achieved an average gain error within |0.466| dBm compared to manual tracking, confirming its orbital prediction accuracy. For the low Earth orbit satellite ARIRANG-5, the system maintained stable signal waveforms throughout the tracking schedule without manual intervention, verifying its continuous tracking capability. Additionally, through simulation experiments, the presented gimbal lock avoidance algorithm successfully tracked 11,469 satellites experiencing gimbal lock within an azimuthal angular velocity limit of 15°/s, demonstrating effective resolution of the singularity problem. Furthermore, the antenna specification reflection function experiment confirmed that tracking errors observed without hardware specification were fully eliminated upon applying the function, verified across both 4 m and 7 m antenna configurations, demonstrating the generality of the presented software across diverse antenna environments. These results confirm that the presented software can provide precise and continuous satellite tracking, significantly improving the reliability and efficiency of satellite data communication. Future research will focus on further enhancing the system’s performance through additional experiments across diverse orbital environments and optimization of the satellite tracking algorithms.

## Figures and Tables

**Table 1:** Comparison between related works and the presented system.

Related Work	Strengths	Limitations	Accuracy	Continuity	Flexibility
C. CHO et al. [7]	low-cost two-axis system	requires manual initialization, high possibility of side-lobe misalignment	Low	Not Supported	Not Supported
Thejaswini M. N. et al. [8]	TLE + SGP4-based orbit prediction	unable to resolve gimbal lock	High	Limited	Not Supported
Oguz K. H. et al. [9]	four-axis antenna-based gimbal lock solution	high system complexity, insufficient algorithm validation	Low	Supported	Not Supported
NSTracker	TLE + SGP4 precise prediction, three-axis gimbal lock avoidance, hardware adaptability	-	High	Supported	Supported

**Table 2:** Mechanical and motion specifications of the three-axis parabolic antenna.

Antenna Model	3-axis Parabolic Antenna
Tilt Angle	East, $7^{\circ }$
Azimuth Rotation Range	$-270^{\circ }$~$270^{\circ }$
Elevation Rotation Range	$0^{\circ }$~$90^{\circ }$
Train Rotation Range	$-270^{\circ }$~$270^{\circ }$
Maximum Azimuth Angular Velocity	15°/s
Maximum Elevation Angular Velocity	10°/s
Maximum Train Angular Velocity	5°/s
Ground-to-Tilt Axis Distance	1.72 m
Tilt-to-Azimuth Axis Distance	0.29 m
Azimuth-to-Elevation Axis Distance	1.24 m

**Table 3:** Azimuth and elevation errors in the solar tracking experiment.

Time	Azimuth (°)	Elevation (°)	Azimuth Error (°)	Elevation Error (°)
09:31	−57.42	30.64	−0.3	0.1
10:25	−46.13	40.40	0	0.1
11:13	−33.24	48.09	0	0
11:55	−18.59	53.46	0.2	0.02
13:30	22.80	56.79	0	0
14:33	48.18	48.19	−0.1	0.1
15:24	63.62	44.10	−0.2	0.15
16:32	78.61	31.94	−0.3	0.1
17:05	85.17	24.76	−0.3	0.1

**Table 4:** TLE of GEO-KOMPSAT-2A.

1 43823U 18100A 24063.77203524 −.00000337 00000+0 00000+0 0 9997
2 43823 0.0338 17.7155 0001576 35.5555 154.9306 1.00271926 19252

**Table 5:** TLE of ARIRANG-5.

1 39227U 13042A 24089.16051463 .00002581 00000+0 18881−3 0 9996
2 39227 97.6203 276.1802 0001457 51.4683 308.6674 15.04482936582126

**Table 6:** Arirang-5 Tracking Result.

Date	18 March 2024	19 March 2024	20 March 2024
Result	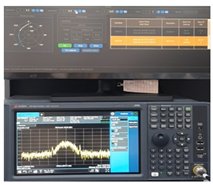	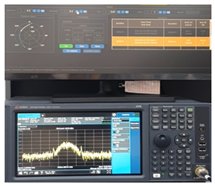	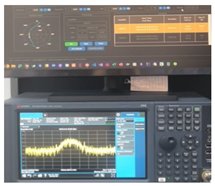
Dropout Count	0	0	0
Maximum Elevation (°)	85.24	75.5	80.38
Duration (min)	12.53	12.30	13.73
Max Gain (dbm)	−45.3	−46.2	−45.4

**Table 7:** Gimbal lock simulation experiment result.

No	Total	Gimbal Lock	Max Speed (°/s)		Over Schedule
			2-Axis	3-Axis	
1	9753	597	2248.77	14.41	0
2	9771	639	1952.26	11.76	0
3	9763	599	2223.99	12.06	0
4	9770	632	2092.53	13.76	0
5	9791	660	2618.88	9.69	0
6	9795	661	2122.54	11.45	0
7	9829	647	1737.98	11.80	0
8	9829	603	1984.76	12.46	0
9	9868	665	1944.18	11.21	0
10	9865	620	1805.31	12.31	0
11	9865	681	1874.51	12.19	0
12	9906	664	2569.73	12.52	0
13	9906	616	1349.25	10.67	0
14	9889	650	2224.59	13.61	0
15	9889	644	2513.01	11.89	0
16	9886	628	2324.32	14.58	0
17	9885	622	2484.63	10.02	0
18	9892	641	1984.81	11.00	0

**Table 8:** Effect of antenna specification on tracking performance.

Metric	4 m Antenna		7 m Antenna	
	Spec O	Spec X	Spec O	Spec X
Azimuth Offset (°)	0	0.1	0	0.2
Elevation Offset (°)	0	0.2	0	0.2
Gain (dBm)	−58.41	−58.66	−67.45	−67.12

## Data Availability

No new datasets were created in this study. The Two-Line Element (TLE) data used for GEO-KOMPSAT-2 and ARIRANG-5 were obtained from the publicly accessible CelesTrak database (https://celestrak.org/). The specific TLE data used in the experiments are provided within the article.
